# Machine learning diagnostic model integrating ultrasound features and immune-inflammatory biomarkers for identifying papillary thyroid carcinoma in patients with Hashimoto’s thyroiditis

**DOI:** 10.3389/fimmu.2026.1902420

**Published:** 2026-09-16

**Authors:** Lulu Hu, Lu Li, Fengsheng Zhou, Yunfeng Chen

**Affiliations:** 1 Department of Ultrasound, the Affiliated Wuxi People's Hospital of Nanjing Medical University; Wuxi People's Hospital; Wuxi Medical Center, Nanjing Medical University, Wuxi, China; 2 Department of Pathology, The First People's Hospital of Chenzhou, Chenzhou, China; 3 Department of Ultrasound, Nanjing Gaochun People's Hospital, Nanjing, China

**Keywords:** Hashimoto’s thyroiditis, immune-inflammatory biomarkers, machine learning, papillary thyroid carcinoma, risk calculator, SHAP, ultrasound

## Abstract

**Background:**

Differentiating papillary thyroid carcinoma (PTC) from benign nodules in patients with Hashimoto’s thyroiditis (HT) remains a significant clinical challenge due to the complex sonographic background and overlapping inflammatory features. This study aimed to develop and validate an interpretable machine learning (ML) model integrating ultrasound features and immune-inflammatory biomarkers for PTC identification in HT patients.

**Methods:**

A multicenter retrospective study was conducted, enrolling 1, 200 HT patients with thyroid nodules from Wuxi People’s Hospital (January 2020 to December 2025), randomly divided into a training cohort (n=840) and a testing cohort (n=360). An independent external validation cohort (n=550) was recruited from Nanjing Gaochun People’s Hospital during the same period. A total of 38 candidate variables, including clinical characteristics, ultrasound features, and immune-inflammatory indices, were evaluated. Least Absolute Shrinkage and Selection Operator (LASSO) and multivariate logistic regression were utilized for feature selection. Seven ML algorithms were compared to construct the optimal predictive model. Model performance was evaluated using the area under the receiver operating characteristic curve (AUC), calibration curves, and decision curve analysis (DCA). SHapley Additive exPlanations (SHAP) were employed to enhance model interpretability.

**Results:**

Six variables were selected as the most informative features for predicting the pathological outcome of PTC: Systemic Immune-Inflammation Index (SII), Thyroid Peroxidase Antibody (TPOAb), pseudonodule formation, shear wave elastography maximum elasticity (SWE-Emax), vascularity grade, and TI-RADS category. The Random Forest (RF) model demonstrated superior performance, achieving an AUC of 0.907 in the training cohort, 0.872 in the testing cohort, and 0.823 in the external validation cohort. Calibration curves and DCA confirmed the model’s high clinical net benefit. SHAP analysis revealed that SII, TPOAb, and pseudonodule formation were the top three contributing features. A web-based risk calculator was subsequently deployed for clinical application.

**Conclusion:**

The proposed interpretable RF model effectively integrates ultrasound features and immune-inflammatory biomarkers to accurately identify PTC in HT patients. The developed web-based calculator provides a practical, non-invasive tool to assist clinicians in personalized decision-making.

## 1 Introduction

Thyroid cancer is the most common endocrine malignancy, with papillary thyroid carcinoma (PTC) accounting for the vast majority of cases (1). Hashimoto’s thyroiditis (HT), the most prevalent autoimmune thyroid disease, is frequently coexistent with PTC (2). Epidemiological and pathological studies have established a strong association between HT and PTC (3). However, the accurate preoperative diagnosis of PTC in the context of HT remains a formidable clinical challenge. The heterogeneous echotexture, diffuse parenchymal changes, and frequent presence of “pseudonodules” in HT glands may complicate the sonographic assessment of true malignant nodules and may be associated with false-positive findings, unnecessary fine-needle aspiration (FNA) biopsies, and diagnostic surgeries (4, 5).

Currently, the American College of Radiology Thyroid Imaging, Reporting and Data System (ACR TI-RADS) and FNA are the cornerstones of thyroid nodule management (6). Nevertheless, the diagnostic performance of TI-RADS may be reduced in patients with HT. Furthermore, FNA yields a high rate of indeterminate results (Bethesda III/IV) in HT patients due to the abundance of lymphocytes and Hurthle cells, complicating clinical decision-making. Therefore, there is an urgent need for a non-invasive, highly accurate, and comprehensive diagnostic tool tailored for this specific population.

Recent evidence suggests that peripheral immune-inflammatory markers are associated with the presence, progression, or prognosis of thyroid cancer (7). Biomarkers derived from routine blood tests, such as the Systemic Immune-Inflammation Index (SII), Systemic Inflammation Response Index (SIRI), and Neutrophil-to-Lymphocyte Ratio (NLR), have shown promising prognostic and diagnostic value in various malignancies (8). Integrating these readily available, cost-effective peripheral markers with high-resolution ultrasound features may provide complementary information for identifying PTC in patients with HT.

Machine learning (ML) has been increasingly applied to medical image analysis and clinical predictive modeling because it can model complex, potentially nonlinear relationships among multiple variables (9). However, the “black-box” nature of many ML algorithms hinders their clinical adoption (10). The advent of SHapley Additive exPlanations (SHAP) provides a robust mathematical framework to interpret ML predictions, thereby bridging the gap between computational power and clinical trust (11).

Recent machine-learning studies have emphasized the value of integrating multiple complementary feature representations for biological classification. For example, the scMFF framework used multiple feature-fusion strategies to improve cell-type identification, highlighting the potential of combining heterogeneous information sources to capture complex biological patterns (12). Inspired by this concept, the present study integrates ultrasound characteristics with peripheral immune-inflammatory biomarkers to provide complementary information for identifying PTC in patients with HT.

To date, no study has comprehensively integrated advanced ultrasound parameters, systemic immune-inflammatory biomarkers, and interpretable ML techniques to diagnose PTC specifically in HT patients (13). Therefore, this multicenter study aimed to: (1) identify the most predictive ultrasound and immune-inflammatory features for PTC in HT; (2) develop, compare, and validate an optimal, interpretable ML diagnostic model across large training, testing, and external validation cohorts; and (3) translate the optimal model into a user-friendly web-based calculator to facilitate individualized clinical decision-making.

## 2 Methods

### 2.1 Study design and patient population

This multicenter retrospective study was conducted in accordance with the Declaration of Helsinki and approved by the Institutional Review Boards of the Affiliated Wuxi People’s Hospital of Nanjing Medical University. The requirement for informed consent was waived due to the retrospective nature of the study.

A total of 1, 200 consecutive patients diagnosed with HT and concomitant thyroid nodules were recruited from Wuxi People’s Hospital between January 1, 2020, and December 31, 2025. These patients were randomly divided into a training cohort (n = 840, 70%) and an internal testing cohort (n = 360, 30%) using a 7:3 ratio. To evaluate the generalizability of the model, an independent external validation cohort comprising 550 HT patients with thyroid nodules was collected from Nanjing Gaochun People’s Hospital during the identical time frame (January 1, 2020, to December 31, 2025). Two radiologists independently evaluated the ultrasound features while blinded to pathological results. Disagreements were resolved by consensus or senior-radiologist adjudication. For SWE, five measurements were obtained using a circular ROI with a diameter of 3-5mm, adjusted according to the size of the solid component of each nodule. The ROI was placed while avoiding cystic areas, calcifications, artifacts, and the lesion margin. SWE-Emax was defined as the maximum valid elasticity value among these measurements.KNN imputation, LASSO feature selection, and hyperparameter tuning were conducted within the training data of each cross-validation fold to prevent information leakage. The testing and external validation cohorts were not used during preprocessing, feature selection, model fitting, or hyperparameter optimization. The six variables retained after LASSO and multivariable logistic regression were used to construct the parsimonious RF model. Model selection was based on discrimination, calibration, clinical net benefit, and predictor parsimony. Performance was reported using AUC, accuracy, sensitivity, specificity, precision, NPV, F1-score, calibration measures, and 95% CIs. SHAP analysis was performed after model fitting to quantify feature contributions and was not used for feature selection.

#### 2.1.1 Inclusion criteria

(1) age ≥ 18 years; (2) confirmed diagnosis of HT based on serological evidence (elevated TPOAb and/or TgAb) and characteristic sonographic findings; (3) presence of at least one distinct thyroid nodule with a maximum diameter ≥ 5 mm; (4) definitive histopathological diagnosis obtained via surgery or ultrasound-guided core needle biopsy.

#### 2.1.2 Exclusion criteria

(1) a history of other malignancies; (2) concurrent acute or chronic systemic infection or hematological disorders that could affect peripheral blood inflammatory indices; (3) incomplete clinical, laboratory, or ultrasound data; and (4) nodules with purely cystic components. Hashimoto’s thyroiditis was present in all participants. Other coexisting autoimmune diseases were recorded as baseline clinical variables and were included among the initial candidate predictors.

### 2.2 Diagnostic criteria for Hashimoto’s thyroiditis

HT was diagnosed based on elevated serum TPOAb and/or TgAb levels above the assay-specific upper reference limits of the participating laboratory, together with characteristic diffuse sonographic changes, including diffuse or patchy hypoechogenicity, heterogeneous echotexture, and micronodular or grid-like parenchymal changes. The diagnostic criteria were applied consistently at both centers.

### 2.3 Pathological reference standard and index nodule selection

The pathological diagnosis of the index nodule served as the reference standard. PTC was defined by histopathological confirmation of papillary thyroid carcinoma following surgical resection or ultrasound-guided core needle biopsy. The benign group comprised nodules with a definitive benign pathological diagnosis, including nodular hyperplasia, adenomatous nodule, follicular adenoma, or other non-malignant thyroid lesions. Cytologically indeterminate nodules without definitive histopathological confirmation were not classified as benign and were excluded.

One index nodule was included for each patient. In patients with a solitary nodule, that lesion was designated as the index nodule. In patients with multiple nodules, the index nodule was defined as the nodule selected for biopsy or surgery based on the highest sonographic suspicion before the pathological result was known. When multiple nodules had the same TI-RADS category, the largest nodule was selected. Lesion laterality, anatomical location, size, and procedural or surgical records were used for imaging-pathology matching. Only one index nodule was included per patient.

### 2.4 Data collection and variable definition

Clinical data, including age, sex, and body mass index (BMI), were extracted from the electronic medical record system. Preoperative thyroid function tests and complete blood counts were performed within one week prior to the ultrasound examination.

A total of 39 variables were coded in the analytical dataset, consisting of one binary outcome variable (X0, PTC status) and 38 candidate predictor variables (X1–X38), including clinical characteristics, ultrasound features, thyroid-related laboratory variables, and immune-inflammatory indices (detailed in **Table 1**). Immune-inflammatory biomarkers were calculated using the following formulas:NLR = Neutrophil count/Lymphocyte count. PLR = Platelet count/Lymphocyte count. LMR = lymphocyte count/monocyte count. SII = (Platelet count × Neutrophil count)/Lymphocyte count. SIRI = (Monocyte count × Neutrophil count)/Lymphocyte count. AISI = platelet count × neutrophil count × monocyte count/lymphocyte count (14).

**Table 1:** Variable assignments.

No.	Full variable name	Type	Coding/assignment rule
X_0_	Papillary Thyroid Carcinoma (Outcome)	Binary	0 = No (Benign); 1 = Yes (PTC)
X_1_	Age	Continuous	Original value (Years)
X_2_	Gender	Binary	0 = Female; 1 = Male
X_3_	Body Mass Index (BMI)	Continuous	Original value (kg/m²)
X_4_	Family history of thyroid cancer	Binary	0 = No; 1 = Yes
X_5_	Coexisting autoimmune diseases	Binary	0 = No; 1 = Yes
X_6_	Nodule maximum diameter	Continuous	Original value (mm)
X_7_	Nodule location	Categorical	0 = Left Lobe; 1 = Right Lobe; 2 = Isthmus
X_8_	Multiple nodules	Binary	0 = No (Single); 1 = Yes (Multiple)
X_9_	Composition (Solid)	Binary	0 = No (Cystic/Mixed); 1 = Yes (Solid)
X_10_	Echogenicity	Categorical	0 = Isoechoic/Hyperechoic; 1 = Hypoechoic; 2 = Markedly Hypoechoic
X_11_	Echotexture (Heterogeneous)	Binary	0 = No (Homogeneous); 1 = Yes (Heterogeneous)
X_12_	Shape (Irregular)	Binary	0 = No (Regular); 1 = Yes (Irregular)
X_13_	Margin (Ill-defined)	Binary	0 = No (Well-defined); 1 = Yes (Ill-defined)
X_14_	Edge features (Lobulated/Spiculated)	Binary	0 = No; 1 = Yes
X_15_	Taller-than-wide (A/T > 1)	Binary	0 = No (≤ 1); 1 = Yes (> 1)
X_16_	Microcalcification	Binary	0 = No; 1 = Yes
X_17_	Posterior acoustic shadowing	Binary	0 = No; 1 = Yes
X_18_	Vascularity Grade (Adler ≥ II)	Binary	0 = No (Grade 0-I); 1 = Yes (Grade II-III)
X_19_	Suspected capsule invasion	Binary	0 = No; 1 = Yes
X_20_	Elasticity score (Hard, 4-5)	Binary	0 = No (Score 1-3); 1 = Yes (Score 4-5)
X_21_	Maximum shear wave elasticity (SWE-Emax)	Continuous	Original value (kPa)
X_22_	TI-RADS Category (4-5)	Binary	0 = No (Category 3); 1 = Yes (Category 4-5)
X_23_	Background Hypoechogenicity	Binary	0 = None/Mild; 1 = Moderate/Severe
X_24_	Background Heterogeneity	Binary	0 = None/Mild; 1 = Moderate/Severe
X_25_	Grid-like/Micronodular change	Binary	0 = No; 1 = Yes
X_26_	Pseudonodule formation	Binary	0 = No; 1 = Yes
X_27_	Background vascularity	Binary	0 = Normal/Mild; 1 = Markedly increased
X_28_	Isthmus thickness	Continuous	Original value (mm)
X_29_	TPOAb	Continuous	Original value (IU/mL)
X_30_	TgAb	Continuous	Original value (IU/mL)
X_31_	TSH	Continuous	Original value (mIU/L)
X_32_	Free T4	Continuous	Original value (pmol/L)
X_33_	NLR	Continuous	Original value (Ratio)
X_34_	PLR	Continuous	Original value (Ratio)
X_35_	LMR	Continuous	Original value (Ratio)
X_36_	SII	Continuous	Original value
X_37_	SIRI	Continuous	Original value
X_38_	AISI	Continuous	Original value

### 2.5 Ultrasound examination and image analysis

All ultrasound examinations were performed using high-end diagnostic ultrasound systems equipped with high-frequency linear array transducers (4–15 MHz). The examinations were conducted by two board-certified radiologists with over 5 years of experience in thyroid imaging, who were blinded to the pathological results.

Conventional B-mode ultrasound was used to evaluate nodule characteristics (composition, echogenicity, shape, margin, and echogenic foci) and background thyroid parenchyma (diffuse heterogeneity, pseudonodule formation, and cervical lymphadenopathy). The nodules were categorized according to the ACR TI-RADS. Color Doppler flow imaging (CDFI) was utilized to assess vascularity grade (Grade I: absent/minimal flow; Grade II: peripheral flow; Grade III: central/mixed flow).

Shear Wave Elastography (SWE) was subsequently performed to measure tissue stiffness. The maximum elasticity value (SWE-Emax, expressed in kilopascals, kPa) of the nodule and the surrounding parenchyma was recorded after a stable color map was obtained.

### 2.6 Feature selection and model construction

Data preprocessing was first conducted to evaluate missing values. As the missing rate for all 38 variables was strictly controlled ≤ 5% (**Table 2**), missing data were imputed using the k-nearest neighbors (KNN) algorithm.

**Table 2:** Missing data rates for candidate variables across the training, testing, and validation cohorts.

Variable	Training cohort (N = 840)	Testing cohort (N = 360)	Validation cohort (N = 550)
Age	0 (0.0%)	0 (0.0%)	0 (0.0%)
Gender	0 (0.0%)	0 (0.0%)	0 (0.0%)
Body Mass Index (BMI)	8 (1.0%)	3 (0.8%)	5 (0.9%)
Family history of thyroid cancer	7 (0.8%)	3 (0.8%)	4 (0.7%)
Coexisting autoimmune diseases	0 (0.0%)	0 (0.0%)	0 (0.0%)
Nodule maximum diameter	13 (1.5%)	5 (1.4%)	8 (1.5%)
Nodule location	0 (0.0%)	0 (0.0%)	0 (0.0%)
Multiple nodules	0 (0.0%)	0 (0.0%)	0 (0.0%)
Composition (Solid)	10 (1.2%)	4 (1.1%)	6 (1.1%)
Echogenicity	0 (0.0%)	0 (0.0%)	0 (0.0%)
Echotexture (Heterogeneous)	0 (0.0%)	0 (0.0%)	0 (0.0%)
Shape (Irregular)	14 (1.7%)	6 (1.7%)	9 (1.6%)
Margin (Ill-defined)	0 (0.0%)	0 (0.0%)	0 (0.0%)
Edge features (Lobulated/Spiculated)	0 (0.0%)	0 (0.0%)	0 (0.0%)
Taller-than-wide (A/T > 1)	0 (0.0%)	0 (0.0%)	0 (0.0%)
Microcalcification	16 (1.9%)	7 (1.9%)	11 (2.0%)
Posterior acoustic shadowing	0 (0.0%)	0 (0.0%)	0 (0.0%)
Vascularity Grade (Adler ≥II)	18 (2.1%)	8 (2.2%)	12 (2.2%)
Suspected capsule invasion	0 (0.0%)	0 (0.0%)	0 (0.0%)
Elasticity score (Hard, 4-5)	20 (2.4%)	9 (2.5%)	13 (2.4%)
Maximum shear wave elasticity (SWE-Emax)	25 (3.0%)	11 (3.1%)	17 (3.1%)
TI-RADS Category (4-5)	0 (0.0%)	0 (0.0%)	0 (0.0%)
Background Hypoechogenicity	0 (0.0%)	0 (0.0%)	0 (0.0%)
Background Heterogeneity	21 (2.5%)	9 (2.5%)	14 (2.5%)
Grid-like/Micronodular change	0 (0.0%)	0 (0.0%)	0 (0.0%)
Pseudonodule formation	0 (0.0%)	0 (0.0%)	0 (0.0%)
Background vascularity	19 (2.3%)	8 (2.2%)	12 (2.2%)
Isthmus thickness	22 (2.6%)	9 (2.5%)	14 (2.5%)
TPOAb	26 (3.1%)	11 (3.1%)	17 (3.1%)
TgAb	28 (3.3%)	12 (3.3%)	18 (3.3%)
TSH	30 (3.6%)	13 (3.6%)	20 (3.6%)
Free T4	32 (3.8%)	14 (3.9%)	21 (3.8%)
NLR	35 (4.2%)	15 (4.2%)	23 (4.2%)
PLR	37 (4.4%)	16 (4.4%)	24 (4.4%)
LMR	33 (3.9%)	14 (3.9%)	22 (4.0%)
SII	39 (4.6%)	17 (4.7%)	26 (4.7%)
SIRI	41 (4.9%)	18 (5.0%)	27 (4.9%)
AISI	40 (4.8%)	17 (4.7%)	26 (4.7%)

Data are presented as Missing Count (Missing Percentage). Missingness was evaluated separately across the three independent cohorts. Imputation parameters were strictly calculated from the Training cohort to prevent data leakage. Variables with 0.0% indicate completely recorded data. All candidate variables had missing rates well ≤ the 5% threshold, justifying the imputation strategy.

Feature selection was performed in a two-step process on the training cohort (**Figure 1**). First, Least Absolute Shrinkage and Selection Operator (LASSO) regression with 10-fold cross-validation was applied to penalize and shrink the coefficients of less important variables. The optimal penalty parameter (λ) was selected using the “one standard error” (1se) rule. Second, the retained features were entered into a multivariate logistic regression model to identify independent predictors.

**Figure 1:**
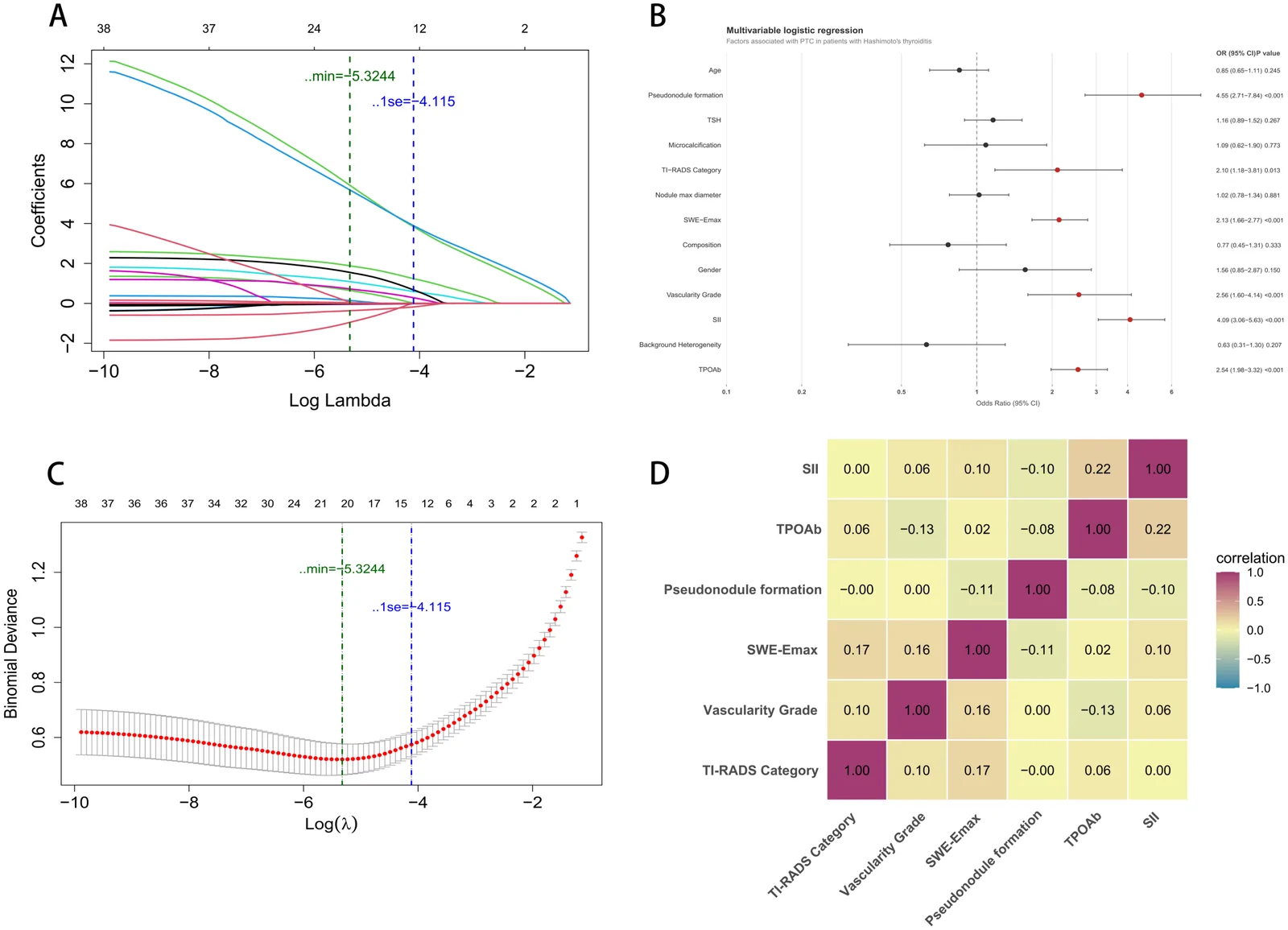
Variable selection and correlation analysis. **(A)** LASSO coefficient profiles for the 38 candidate variables. **(B)** Variables retained at the λ1se value were entered into a multivariable logistic regression model, which identified six independent predictors of papillary thyroid carcinoma (PTC): systemic immune-inflammation index (SII), thyroid peroxidase antibody (TPOAb), pseudonodule formation, maximum shear-wave elasticity (SWE-Emax), vascularity grade, and TI-RADS category. These six variables were subsequently used to construct the parsimonious random forest (RF) model. **(C)** Ten-fold cross-validation for selection of the LASSO penalty parameter; the vertical dashed line indicates the λ1se value. **(D)** Spearman correlation heatmap of the six final predictors.

Seven machine learning algorithms were trained and compared to construct the predictive model: Decision Tree (DT), K-Nearest Neighbors (KNN), Light Gradient Boosting Machine (LGBM), Logistic Regression (LR), Random Forest (RF), Support Vector Machine (SVM), Extreme Gradient Boosting (XGB). Hyperparameter tuning was performed using grid search with 5-fold cross-validation on the training set.

### 2.7 Model evaluation and interpretability

The predictive performance of the models was primarily evaluated using the area under the receiver operating characteristic curve (AUC). Additional metrics, including accuracy, sensitivity, specificity, precision, and F1-score, were calculated. Calibration curves were plotted to assess the agreement between predicted probabilities and actual observed outcomes. Decision Curve Analysis (DCA) and Clinical Impact Curves (CIC) were generated to quantify the clinical net benefit of the optimal model across various threshold probabilities.

To demystify the “black-box” nature of the optimal ML model, SHapley Additive exPlanations (SHAP) were employed (**Figure 2**). SHAP summary plots and dependence plots were generated to visualize the global and local feature importance, illustrating how each predictor positively or negatively influenced the model’s output.

**Figure 2:**
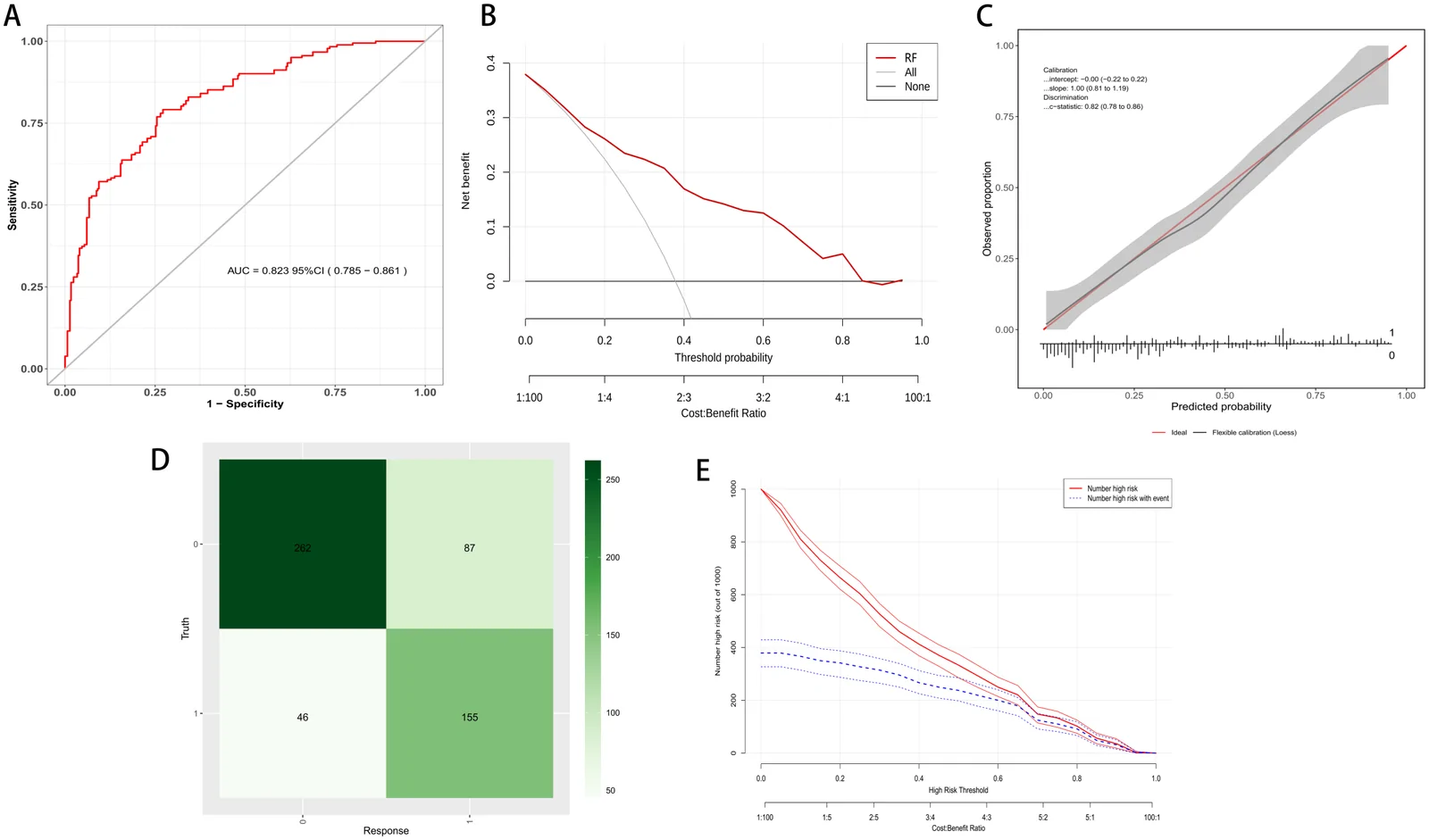
SHAP-based interpretation of the RF model. **(A)** Global feature-importance ranking based on the mean absolute SHAP value. **(B)** SHAP summary plot showing the direction and magnitude of each feature’s contribution to the model output; positive SHAP values indicate an increased predicted probability of PTC. **(C)** SHAP waterfall plot for a representative correctly classified PTC case, showing how individual predictors shifted the prediction from the baseline value to the final model output. **(D)** SHAP waterfall plot for a representative correctly classified benign case, showing the feature contributions that shifted the prediction toward the negative class. SHAP, SHapley Additive exPlanations; RF, random forest; PTC, papillary thyroid carcinoma; SII, systemic immune-inflammation index; TPOAb, thyroid peroxidase antibody; SWE-Emax, maximum shear-wave elasticity.

### 2.8 Clinical translation: web-based risk calculator

To facilitate the clinical translation of the optimal model, a dynamic, user-friendly web-based risk calculator was developed using the Shiny framework in R and deployed online (**Figure 3**). The calculator allows clinicians to input patient-specific variables and instantly obtain the predicted probability of PTC, accompanied by a risk stratification recommendation.

**Figure 3:**
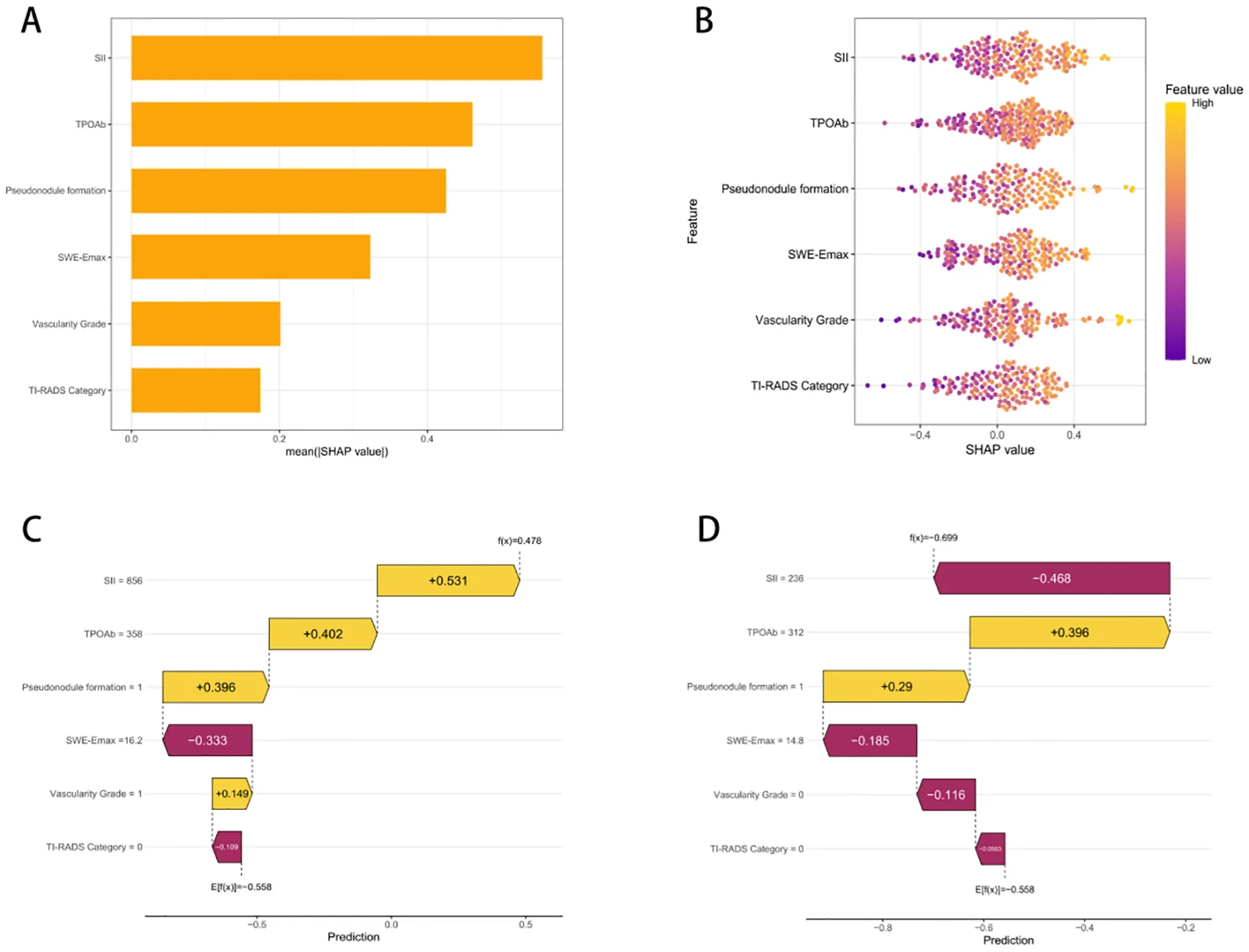
Web-based PTC risk calculator and illustrative cases. The calculator estimates the probability of PTC using SII, TPOAb, SWE-Emax, pseudonodule formation, vascularity grade, and TI-RADS category. **(A)** Representative low-risk example with a predicted PTC probability of 35.6%. **(B)** Representative high-risk example with a predicted PTC probability of 63.4%.

### 2.9 Statistical analysis

Continuous variables were expressed as mean ± standard deviation (SD) or median with interquartile range (IQR), and compared using the Student’s t-test or Mann-Whitney U test, respectively. Categorical variables were presented as frequencies and percentages, and compared using the Chi-square test or Fisher’s exact test. All statistical analyses and ML modeling were performed using R (version 4.2.0). A two-sided *p*-value < 0.05 was considered statistically significant.

## 3 Results

### 3.1 Patient characteristics and data quality

A total of 1, 750 patients with HT and concomitant thyroid nodules were included in this multicenter study. The primary cohort from the Affiliated Wuxi People’s Hospital of Nanjing Medical University (n = 1, 200) was randomly divided into a training cohort (n = 840) and a testing cohort (n = 360) at a 7:3 ratio. An independent external validation cohort (n = 550) was recruited from Nanjing Gaochun People’s Hospital. The prevalence of PTC was consistent across the three cohorts: 35.5% (298/840) in the training set, 33.9% (122/360) in the testing set, and 36.5% (201/550) in the validation set. All 1, 750 included patients had a definitive pathological diagnosis for the index nodule. The training cohort comprised 298 PTC cases and 542 benign cases, the internal testing cohort comprised 122 PTC cases and 238 benign cases, and the external validation cohort comprised 201 PTC cases and 349 benign cases.

As detailed in **Table 3**, there were no statistically significant differences in demographic characteristics, ultrasound features, thyroid background characteristics, thyroid function, or immune-inflammatory biomarkers among the three cohorts (all *p* > 0.05), indicating excellent baseline balance and cohort representativeness. Data quality was rigorously controlled; the missing rates for all 38 candidate variables (X_0_-X_38_, **Table 1**) were strictly maintained ≤ 5% (**Table 2**), ensuring the reliability of subsequent KNN imputation and model training.

**Table 3:** Baseline characteristics of patients in the training, testing, and validation cohorts.

Characteristic	Training cohort (n = 840)	Testing cohort (n = 360)	Validation cohort (n = 550)	*p*-value
Papillary Thyroid Carcinoma (PTC)	298 (35.5%)	122 (33.9%)	201 (36.5%)	0.684
Age, years	45.4 ± 11.8	44.1 ± 12.5	46.2 ± 11.2	0.081
Gender (Male)	188 (22.4%)	76 (21.1%)	128 (23.3%)	0.715
BMI, kg/m²	23.6 ± 3.1	24.1 ± 3.4	23.2 ± 2.9	0.094
Family history of thyroid cancer	69 (8.2%)	26 (7.2%)	51 (9.3%)	0.523
Coexisting autoimmune diseases	85 (10.1%)	38 (10.6%)	52 (9.5%)	0.841
Nodule maximum diameter, mm	11.2 ± 6.8	12.5 ± 7.2	10.8 ± 6.1	0.115
Nodule location				0.284
Left Lobe	398 (47.4%)	158 (43.9%)	275 (50.0%)	
Right Lobe	400 (47.6%)	182 (50.6%)	248 (45.1%)	
Isthmus	42 (5.0%)	20 (5.6%)	27 (4.9%)	
Multiple nodules	382 (45.5%)	158 (43.9%)	265 (48.2%)	0.418
Composition (Solid)	592 (70.5%)	245 (68.1%)	401 (72.9%)	0.297
Echogenicity				0.402
Isoechoic/Hyperechoic	165 (19.6%)	82 (22.8%)	101 (18.4%)	
Hypoechoic	525 (62.5%)	215 (59.7%)	345 (62.7%)	
Markedly Hypoechoic	150 (17.9%)	63 (17.5%)	104 (18.9%)	
Echotexture (Heterogeneous)	465 (55.4%)	192 (53.3%)	312 (56.7%)	0.612
Shape (Irregular)	288 (34.3%)	132 (36.7%)	179 (32.5%)	0.385
Margin (Ill-defined)	321 (38.2%)	128 (35.6%)	225 (40.9%)	0.279
Edge features (Lobulated/Spiculated)	358 (42.6%)	145 (40.3%)	251 (45.6%)	0.264
Taller-than-wide (A/T > 1)	205 (24.4%)	95 (26.4%)	124 (22.5%)	0.352
Microcalcification	232 (27.6%)	105 (29.2%)	145 (26.4%)	0.671
Posterior acoustic shadowing	130 (15.5%)	51 (14.2%)	92 (16.7%)	0.589
Vascularity Grade (Adler ≥ II)	335 (39.9%)	152 (42.2%)	205 (37.3%)	0.275
Suspected capsule invasion	182 (21.7%)	72 (20.0%)	131 (23.8%)	0.347
Elasticity score (Hard, 4-5)	315 (37.5%)	148 (41.1%)	201 (36.5%)	0.298
Maximum shear wave elasticity, kPa	43.1 ± 14.5	44.8 ± 15.2	42.5 ± 13.8	0.076
TI-RADS Category (4-5)	652 (77.6%)	275 (76.4%)	438 (79.6%)	0.443
Background Hypoechogenicity (Moderate/Severe)	685 (81.5%)	291 (80.8%)	458 (83.3%)	0.536
Background Heterogeneity (Moderate/Severe)	738 (87.9%)	312 (86.7%)	490 (89.1%)	0.485
Grid-like/Micronodular change	485 (57.7%)	215 (59.7%)	308 (56.0%)	0.507
Pseudonodule formation	255 (30.4%)	101 (28.1%)	175 (31.8%)	0.462
Background vascularity (Markedly increased)	181 (21.5%)	85 (23.6%)	115 (20.9%)	0.573
Isthmus thickness, mm	4.2 ± 1.1	4.4 ± 1.2	4.1 ± 1.0	0.068
TPOAb, IU/mL	285.4 ± 180.2	301.2 ± 195.5	274.8 ± 170.6	0.123
TgAb, IU/mL	225.8 ± 155.4	240.5 ± 168.2	218.6 ± 145.8	0.158
TSH, mIU/L	2.65 ± 1.54	2.81 ± 1.68	2.58 ± 1.45	0.104
Free T4, pmol/L	15.2 ± 2.3	14.9 ± 2.5	15.4 ± 2.2	0.082
NLR (Neutrophil/Lymphocyte)	1.95 ± 0.65	1.90 ± 0.68	2.02 ± 0.61	0.075
PLR (Platelet/Lymphocyte)	132.5 ± 45.2	128.8 ± 48.5	136.2 ± 43.1	0.119
LMR (Lymphocyte/Monocyte)	4.25 ± 1.15	4.38 ± 1.22	4.15 ± 1.10	0.093
SII	455.2 ± 165.8	440.5 ± 172.4	468.4 ± 158.2	0.088
SIRI	0.88 ± 0.32	0.85 ± 0.35	0.91 ± 0.28	0.085
AISI	178.5 ± 75.4	168.2 ± 80.5	185.6 ± 72.1	0.062

Data are presented as mean ± standard deviation for continuous variables and n (%) for categorical variables.

BMI, Body Mass Index; TI-RADS, Thyroid Imaging Reporting and Data System; TPOAb, Thyroid Peroxidase Antibody; TgAb, Thyroglobulin Antibody; TSH, Thyroid Stimulating Hormone; Free T4, Free Thyroxine; NLR, Neutrophil-to-Lymphocyte Ratio; PLR, Platelet-to-Lymphocyte Ratio; LMR, Lymphocyte-to-Monocyte Ratio; SII, Systemic Immune-Inflammation Index; SIRI, Systemic Inflammation Response Index; AISI, Aggregate Index of Systemic Inflammation.

### 3.2 Feature selection

The feature selection process is illustrated in **Figure 1**. Initially, 38 candidate variables were subjected to LASSO regression to prevent overfitting and eliminate collinearity. Using the 1-standard error (1se) rule in 10-fold cross-validation, the optimal penalty parameter (λ) was determined, shrinking the coefficients of less informative variables to zero. The complete list of the 13 LASSO-selected predictors, together with their corresponding penalized coefficients at the selected lambda.1se value, is provided in **Supplementary Table 2**. The retained features were subsequently entered into a multivariate logistic regression model. Ultimately, six independent predictors were identified as having the highest diagnostic value for PTC in HT patients: Systemic Immune-Inflammation Index (SII), Thyroid Peroxidase Antibody (TPOAb), pseudonodule formation, shear wave elastography maximum elasticity (SWE-Emax), vascularity grade, and TI-RADS category. To prevent information leakage, KNN imputation, LASSO feature selection, and hyperparameter tuning were performed exclusively within the training cohort and repeated independently within each cross-validation fold. The finalized pipeline was subsequently refitted using the complete training cohort and applied unchanged to the independent test cohort, which was reserved exclusively for final performance evaluation.

### 3.3 Comparison of random forest models based on different predictor sets

In the training cohort, the apparent performance of three Random Forest models based on 38, 13, and 6 predictors was compared. RF-all achieved the highest AUC and overall classification performance, followed by RF-13 and RF-6. The RF-6 model achieved an AUC of 0.907, an accuracy of 0.81, a sensitivity of 0.87, a specificity of 0.77, a precision of 0.70, an NPV of 0.91, and an F1-score of 0.78 (**Supplementary Table 1**). Despite its slightly lower apparent training performance, RF-6 was selected as the final model because it provided a more parsimonious and clinically applicable predictor set.

### 3.4 Model development and internal validation

Seven machine learning algorithms (DT, KNN, LGBM, LR, RF, SVM, XGB) were trained and evaluated. **Figure 4** illustrates the performance comparison of these models specifically in the training cohort. The Random Forest (RF) model demonstrated superior predictive capability, achieving the highest AUC of 0.907 (95% CI: 0.882–0.932). Comprehensive comparisons of evaluation metrics (accuracy, sensitivity, specificity, F1-score) across all models in the training set further solidified the RF model as the optimal choice.

**Figure 4:**
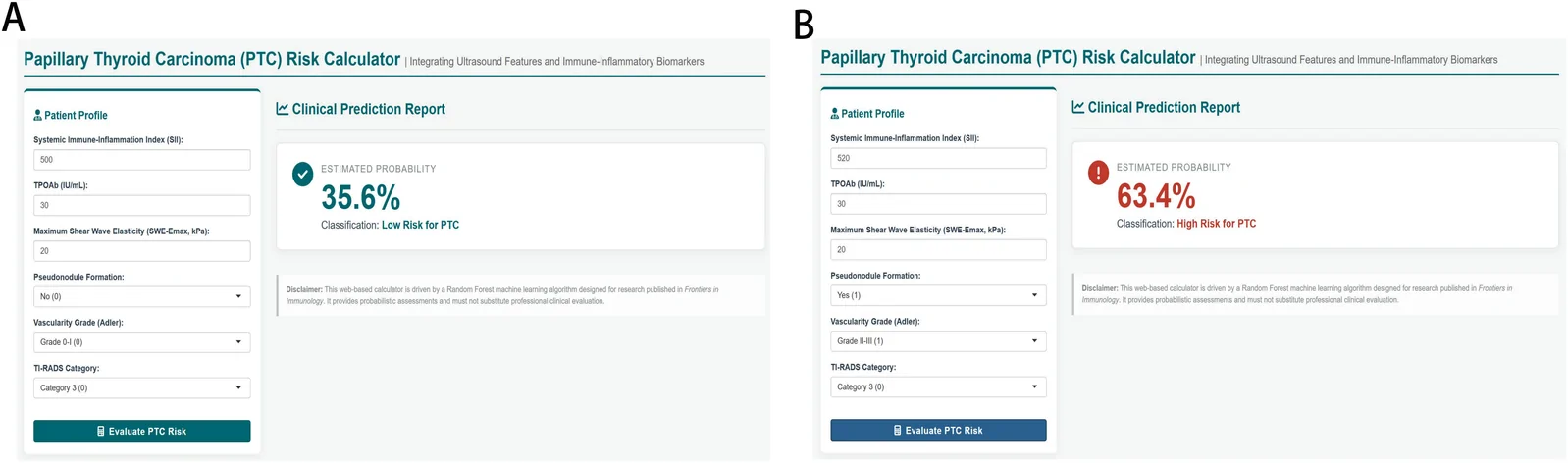
Development and comparison of machine-learning models. **(A)** Receiver operating characteristic (ROC) curves for the seven evaluated models: decision tree (DT), k-nearest neighbors (KNN), light gradient boosting machine (LGBM), logistic regression (LR), random forest (RF), support vector machine (SVM), and extreme gradient boosting (XGB). **(B)** Pairwise comparisons of the areas under the ROC curves (AUCs) between models using the DeLong test for correlated ROC curves. **(C)** Comparison of AUC values across models. **(D)** Heatmap of classification-performance metrics, including accuracy, sensitivity, specificity, precision, and F1-score. **(E)** Decision curve analysis (DCA) showing the net clinical benefit of the models across a range of threshold probabilities. **(F)** Calibration curves showing the agreement between predicted probabilities and observed outcomes. **(G)** Radar chart comparing the principal performance metrics of the evaluated models. **(H)** Precision-recall curves and corresponding areas under the precision-recall curves (PRAUCs).

Subsequently, the optimal RF model was evaluated in the testing cohort for internal validation (**Figure 5**). The RF model maintained robust discriminative performance, yielding an AUC of 0.872 (95% CI: 0.840–0.905). Calibration curves showed excellent agreement between the RF model’s predicted probabilities and actual observed outcomes. Decision Curve Analysis (DCA) confirmed that the RF model provided a higher net clinical benefit across a wide range of threshold probabilities compared to the “treat-all” or “treat-none” strategies.

**Figure 5:**
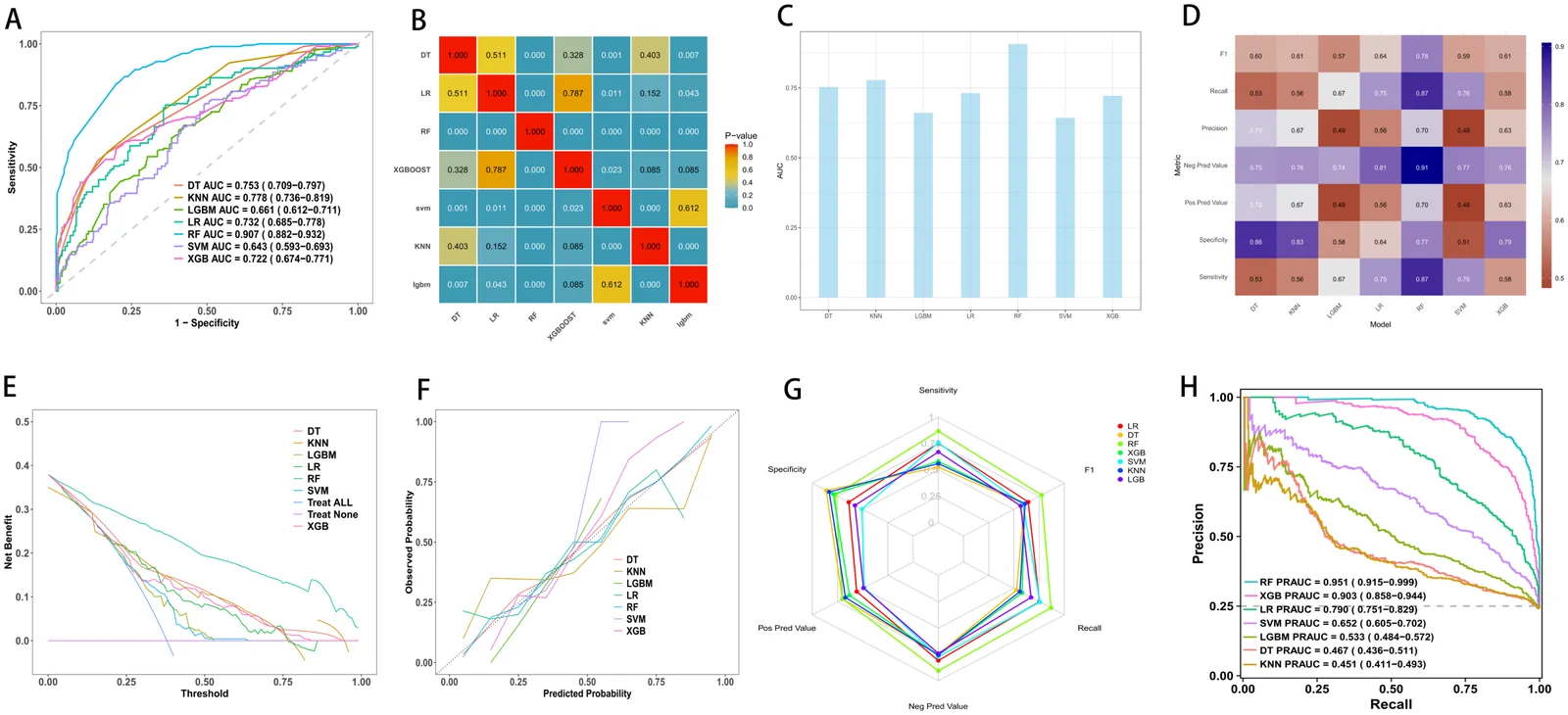
Performance of the RF model in the internal testing cohort. **(A)** ROC curve of the RF model in the internal testing cohort, with an AUC of 0.872 (95% CI, 0.840-0.905). **(B)** Decision curve analysis (DCA) comparing the net clinical benefit of the RF model with the treat-all and treat-none strategies across threshold probabilities. **(C)** Calibration curve showing agreement between predicted and observed probabilities. **(D)** Confusion matrix showing 195 true-negative, 43 false-positive, 24 false-negative, and 98 true-positive classifications. **(E)** Clinical impact curve (CIC) showing the number of individuals classified as high risk and the corresponding number of true-positive events across risk thresholds.

Compared with ACR TI-RADS alone, the RF model showed better discrimination in both the internal testing cohort (AUC, 0.872 vs. 0.742; P < 0.001) and the external validation cohort (AUC, 0.823 vs. 0.716; P < 0.001). The RF model also demonstrated higher sensitivity, specificity, accuracy, and F1-score in both cohorts.

### 3.5 External validation

To evaluate the generalizability of the optimal RF model, it was applied to the independent external validation cohort (**Figure 6**). The RF model maintained strong discriminative performance in this unseen population, yielding an AUC of 0.823 (95% CI: 0.785–0.861). The calibration curve indicated good fitness, and both DCA and Clinical Impact Curves (CIC) demonstrated that the model consistently provided substantial net clinical benefits in the external population, supporting the model’s reproducibility and preliminary generalizability across the participating medical centers.

**Figure 6:**
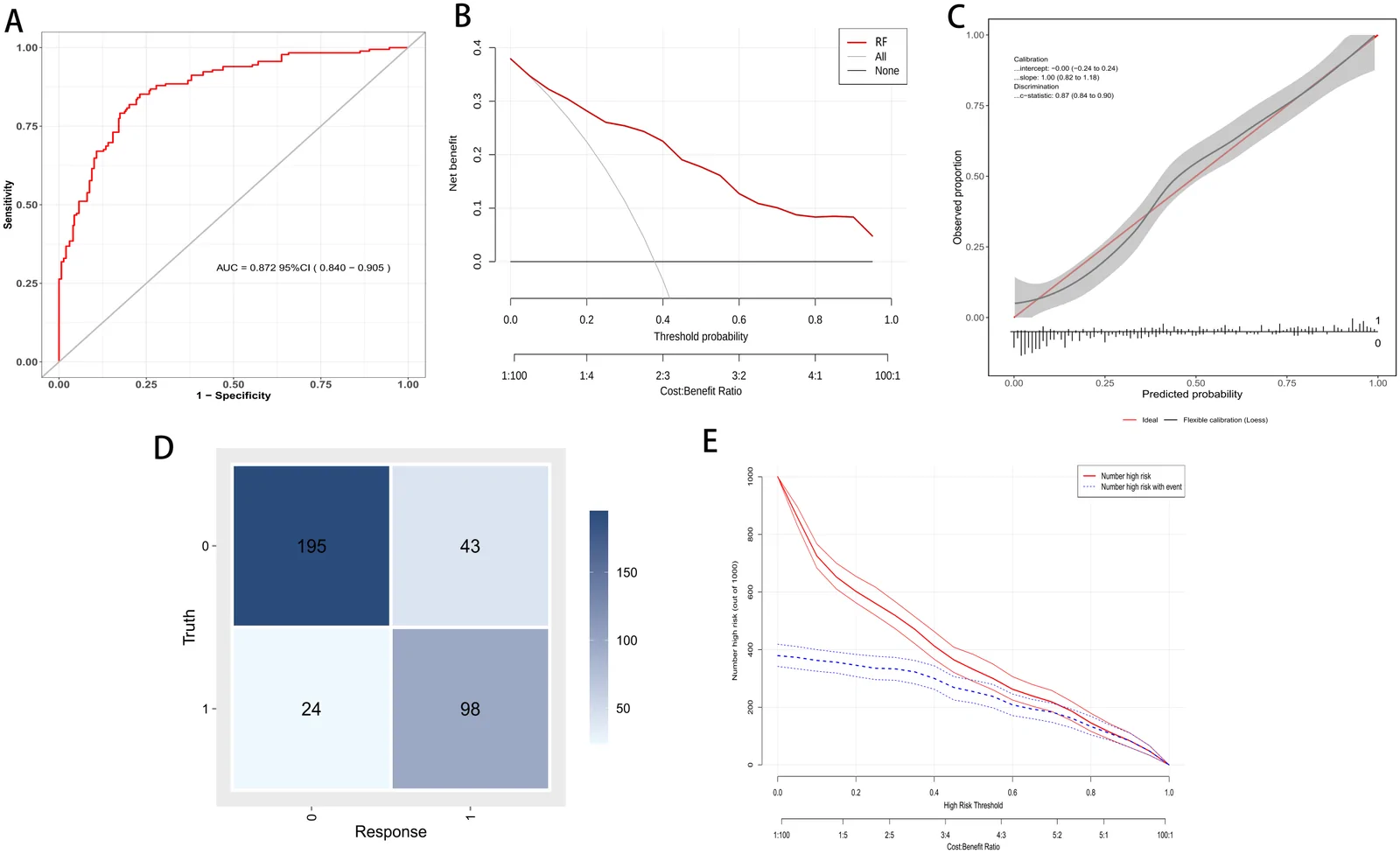
External validation of the RF model. **(A)** ROC curve demonstrating the discrimination of the RF model in the external validation cohort, with an AUC of 0.823 (95% CI, 0.785-0.861). **(B)** Decision curve analysis (DCA) comparing the net clinical benefit of the RF model with the treat-all and treat-none strategies across threshold probabilities. **(C)** Calibration plot showing the agreement between predicted and observed probabilities. **(D)** Confusion matrix showing 262 true-negative, 87 false-positive, 46 false-negative, and 155 true-positive classifications. **(E)** Clinical impact curve (CIC) showing the number of patients classified as high risk and the number of true-positive events per 1, 000 patients at different risk thresholds.

### 3.6 Model interpretability via SHAP

To overcome the “black-box” limitation of the RF model, SHAP analysis was employed to interpret the model’s decision-making process (**Figure 2**). The SHAP summary plot revealed the global importance and directional impact of the six predictors. SII, TPOAb, and pseudonodule formation emerged as the top three most influential features driving the model’s predictions. Specifically, higher SII values, lower TPOAb titers, and the absence of pseudonodule formation were strongly associated with an increased probability of PTC. The SHAP dependence plots further visualized the non-linear relationships and marginal effects of these top features on the model’s output, providing clinicians with transparent, physiologically plausible insights into the risk stratification.

### 3.7 Clinical translation: web-based risk calculator

To facilitate the seamless integration of the RF model into routine clinical practice, a dynamic, web-based PTC risk calculator was developed and deployed (URL: https://my-portfolio.shinyapps.io/Papillary_Thyroid_Carcinoma_Calculator/). As demonstrated in **Figure 3**, the calculator features a user-friendly interface where clinicians can input the six predictor variables. The tool instantly computes the individualized probability of PTC and provides risk stratification. Two illustrative cases are presented: a low-risk patient with a predicted probability of 35.6%, and a high-risk patient with a predicted probability of 63.4%, highlighting its utility in guiding personalized decisions regarding FNA biopsy or active surveillance.

## 4 Discussion

In this multicenter retrospective study, we successfully developed and validated an interpretable machine learning diagnostic model that integrates ultrasound features and immune-inflammatory biomarkers to identify PTC in patients with HT. By leveraging a large cohort of 1, 750 patients across two independent medical centers, our Random Forest model demonstrated good discriminative performance, with an AUC greater than 0.82 in all three cohorts, and showed potential clinical utility based on calibration and decision-curve analyses. Furthermore, SHAP analysis demystified the model’s decision process, and the deployment of a web-based calculator provides a practical, non-invasive tool to assist clinicians in personalized decision-making.

Our findings are also consistent with recent applications of machine learning in biological prediction. A recent study explored potential transcription factors and their regulatory relationships using an asymmetric covariance natural vector encoding method combined with machine-learning algorithms, demonstrating the ability of machine learning to identify complex biological associations (15). Similarly, our model captured nonlinear relationships between ultrasound features and immune-inflammatory biomarkers. However, the predictors identified in our study should be interpreted as clinically useful predictive factors rather than direct evidence of underlying molecular mechanisms.

The coexistence of HT and PTC presents a unique diagnostic dilemma. HT is characterized by diffuse lymphocytic infiltration, fibrosis, and regenerative hyperplasia, and these pathological features may be accompanied by a heterogeneous sonographic background and pseudonodule-like appearances that complicate the assessment of true malignant nodules (16). Consequently, conventional TI-RADS often yields high false-positive rates in this population, leading to unnecessary biopsies (17). Our study addresses this gap by incorporating pseudonodule formation and SWE-Emax as key predictors. In our study, pseudonodule formation was associated with a lower model-predicted probability of malignancy in the assessed nodule. This finding may reflect differences in the sonographic or inflammatory background, but the underlying explanation remains uncertain. SWE-Emax was associated with PTC status and provided information complementary to conventional morphological ultrasound features. Although increased tissue stiffness has been described in some malignant thyroid nodules, our study did not directly assess desmoplastic stromal reactions; therefore, a specific pathological mechanism cannot be inferred from our findings (18, 19).

A notable feature of our model is the inclusion of two peripheral immune-inflammatory variables, SII and TPOAb, in addition to ultrasound characteristics. SII is calculated from peripheral platelet, neutrophil, and lymphocyte counts and has been investigated as a composite marker of systemic inflammatory status in patients with cancer (20–22). In our study, higher SII values were associated with a higher predicted probability of PTC, whereas higher TPOAb levels showed an inverse association with model predictions. These findings suggest that SII and TPOAb may provide diagnostic information complementary to ultrasound features in patients with HT (23, 24). However, neither marker directly measures immune-cell exhaustion, antitumor immune surveillance, immune-cell infiltration, or the local tumor immune microenvironment. Accordingly, the biological basis of these associations remains uncertain, and the observed relationships should not be interpreted as evidence of immune exhaustion or a systemic immune–local tumor axis (25). The inverse association observed between TPOAb and the model output is hypothesis-generating and requires confirmation in prospective studies incorporating longitudinal immune measurements and tissue-based immune profiling.

While machine learning models often suffer from poor clinical adoption due to their “black-box” nature, we employed SHAP to ensure transparency (26). The SHAP analysis confirmed that SII, TPOAb, and pseudonodule formation were the top drivers of the model. This interpretability may facilitate clinical understanding by showing how each included variable contributes to an individual model prediction, without implying a causal or mechanistic relationship. For instance, the SHAP dependence plots illustrated the non-linear association between SII values and the model output within the study population.

To translate these findings into clinical practice, we developed a web-based risk calculator. In the era of precision medicine, such tools are invaluable for shared decision-making. For patients with a high predicted probability, the calculator may provide additional information to support consideration of FNA or other appropriate clinical evaluation. For patients with a lower predicted probability, it may assist clinicians in considering follow-up or active surveillance in conjunction with established clinical guidelines and shared decision-making. Whether use of the calculator reduces unnecessary procedures, healthcare costs, or patient anxiety requires prospective evaluation.

Despite the strengths of our multicenter design and large sample size, several limitations must be acknowledged. First, the retrospective nature of the study may introduce inherent selection bias. Second, although we included an external validation cohort, both centers are located in the same geographic region (Jiangsu Province, China); future prospective, multinational studies are needed to validate the model across diverse ethnic and geographic populations. Third, the ultrasound evaluations and SWE measurements, although performed by experienced radiologists, may still be subject to inter-operator variability. Fourth, the immunological interpretation of our findings is limited because SII and TPOAb are peripheral blood-based markers and do not directly characterize the local tumor immune microenvironment. We did not assess immune-cell phenotypes, exhaustion markers, immune checkpoint expression, cytokine profiles, or immune-cell infiltration in tumor tissue. Therefore, the present findings cannot establish immune exhaustion, antitumor immune surveillance, or a systemic immune–local tumor axis. Moreover, given the retrospective observational design, the associations of SII and TPOAb with PTC prediction should not be interpreted as causal. Future studies integrating longitudinal peripheral immune measurements with tumor-tissue immune profiling are needed to investigate the underlying biological mechanisms. Finally, the model was designed specifically for patients with HT; its performance in patients with other forms of thyroiditis or normal thyroid backgrounds requires further investigation.

## 5 Conclusion

In conclusion, we developed and externally validated an interpretable Random Forest model integrating ultrasound features (pseudonodule formation, SWE-Emax, vascularity grade, and TI-RADS category) with peripheral immune-inflammatory variables (SII and TPOAb) for the preoperative identification of PTC in patients with HT. SHAP analysis provided transparent information on the contribution of each variable to the model predictions but should not be interpreted as evidence of an underlying immunological mechanism. The web-based calculator may serve as a practical, non-invasive clinical decision-support tool for thyroid nodule assessment in patients with HT. Further prospective validation is required to assess its clinical impact and to determine whether its use improves clinical decision-making before routine implementation.

## Data Availability

The raw data supporting the conclusions of this article will be made available by the authors, without undue reservation.
