# Supplementary material for: Machine learning diagnostic model integrating ultrasound features and immune-inflammatory biomarkers for identifying papillary thyroid carcinoma in patients with Hashimoto’s thyroiditis
Source: Front Immunol. 2026 Sep 16;17:1902420. doi: 10.3389/fimmu.2026.1902420 (PMC13624124; doi:10.3389/fimmu.2026.1902420)
Supplement: Supplementary file 1 [file Supplementaryfile1.docx]

**Supplementary Table 1. Comparison of the apparent training performance of Random Forest models based on different predictor sets.**

| **Model** | **Predictor set** | **No. of predictors** | **AUC** | **Accuracy** | **Sensitivity** | **Specificity** | **Precision** | **NPV** | **F1-score** |
| --- | --- | --- | --- | --- | --- | --- | --- | --- | --- |
| RF-all | All candidate predictors | 38 | 0.963 | 0.91 | 0.91 | 0.92 | 0.86 | 0.95 | 0.88 |
| RF-13 | LASSO-selected predictors | 13 | 0.938 | 0.88 | 0.84 | 0.90 | 0.82 | 0.92 | 0.83 |
| RF-6 | Final six predictors | 6 | 0.907 | 0.81 | 0.87 | 0.77 | 0.70 | 0.91 | 0.78 |
