# Supplementary material for: Machine learning diagnostic model integrating ultrasound features and immune-inflammatory biomarkers for identifying papillary thyroid carcinoma in patients with Hashimoto’s thyroiditis
Source: Front Immunol. 2026 Sep 16;17:1902420. doi: 10.3389/fimmu.2026.1902420 (PMC13624124; doi:10.3389/fimmu.2026.1902420)
Supplement: Supplementary file 2 [file Supplementaryfile2.docx]

**Supplementary Table S2. The 13 predictors selected by LASSO logistic regression and their penalized coefficients**

| **No.** | **Predictor** | **Variable type** | **Coding or scale** | **LASSO coefficient** |
| --- | --- | --- | --- | --- |
| 1 | Age | Continuous | Per 1 year | 0.084 |
| 2 | Nodule maximum diameter | Continuous | Per 1 mm | 0.067 |
| 3 | Echogenicity | Categorical | Hypoechoic or markedly hypoechoic vs isoechoic/hyperechoic | 0.218 |
| 4 | Margin | Binary | Ill-defined = 1; well-defined = 0 | 0.146 |
| 5 | Microcalcification | Binary | Yes = 1; no = 0 | 0.193 |
| 6 | Maximum shear wave elasticity (SWE-Emax) | Continuous | Per 1 kPa | 0.126 |
| 7 | TI-RADS category | Binary | Category 4-5 = 1; category 3 = 0 | 0.361 |
| 8 | Pseudonodule formation | Binary | Yes = 1; no = 0 | -0.284 |
| 9 | Vascularity grade | Binary | Grade II-III = 1; grade 0-I = 0 | 0.241 |
| 10 | Thyroid peroxidase antibody (TPOAb) | Continuous | Per 1 standard deviation | -0.317 |
| 11 | Thyroid-stimulating hormone (TSH) | Continuous | Per 1 mIU/L | 0.071 |
| 12 | Neutrophil-to-lymphocyte ratio (NLR) | Continuous | Per 1 standard deviation | 0.109 |
| 13 | Systemic immune-inflammation index (SII) | Continuous | Per 1 standard deviation | 0.438 |
